# Association between nocturnal sleep duration and midday napping and the incidence of sarcopenia in middle-aged and older adults: a 4-year longitudinal study

**DOI:** 10.1265/ehpm.24-00046

**Published:** 2024-05-17

**Authors:** Ji He, Jin Wang, Beibei Pan, Hongjun Zhang, Shaoshuai Shen, Xiaoguang Zhao

**Affiliations:** 1 Faculty of Public Foundation, Taizhou Vocational College of Science & Technology, Taizhou 318020, Zhejiang, China; 2 Faculty of Sports Science, Ningbo University, Ningbo 315211, Zhejiang, China; 3 School of Teacher Education, Taizhou University, Taizhou 318000, Zhejiang, China; 4 School of Physical Education, Liaoning Finance and Trade College, Xingcheng 125100, Liaoning, China; 5 School of Education and Welfare, Aichi Prefectural University, Nagakute, Aichi 480-1198, Japan

**Keywords:** China, Midday napping, Middle-aged and older adults, Sarcopenia, Sleep duration

## Abstract

**Background:**

Identifying treatment targets for sarcopenia is a public health concern. This study aimed to examine the association of nocturnal sleep duration and midday napping with the presence of sarcopenia in middle-aged and older adults, utilizing data from the China Health and Retirement Longitudinal Study in 2011 and 2015.

**Methods:**

A sum of 7,926 individuals (≥40 years) took part in this study. Sarcopenia was diagnosed according to the Asian Working Group for Sarcopenia. A self-reported questionnaire was used to collect data on nocturnal sleep duration and midday napping. Nocturnal sleep duration was categorized into three groups: short sleepers (<6 h), normal sleepers (6–8 h), and long sleepers (>8 h). Midday napping was coded as a dichotomous outcome (yes/no).

**Results:**

The incidence of sarcopenia was 5.3% during the 4-year follow-up. Short sleep duration (<6 h) was substantially linked to an increased incidence of sarcopenia (OR: 1.50, 95% CI: 1.21–1.87) as compared to nocturnal sleep length (6–8 h). Adults with midday napping had a lower risk of developing sarcopenia than non-nappers (OR: 0.78, 95% CI: 0.63–0.95). We further found that short sleepers with midday napping did not have a significantly higher risk of subsequent diagnosis of sarcopenia compared to normal sleepers without midday napping.

**Conclusion:**

These findings imply that short sleep duration in middle-aged and older persons is related to an increased incidence of sarcopenia. However, the adverse effect of short sleep duration on sarcopenia can be compensated by midday napping.

**Supplementary information:**

The online version contains supplementary material available at https://doi.org/10.1265/ehpm.24-00046.

## 1. Background

Sarcopenia is a growing public health concern around the world. It is a common geriatric illness that is defined by a decrease in muscle strength and physical performance as well as an age-related loss of skeletal muscle mass [1]. There is accumulating evidence that sarcopenia is related to a series of negative health outcomes including functional disability, falls, frailty, fractures, morbidity, and mortality [2–4]. Sarcopenia presents a significant challenge to healthy aging. Thus, it is crucial to identify treatment targets for sarcopenia to prevent or delay its onset.

To prevent or delay sarcopenia, a variety of lifestyle behaviors have been comprehensively investigated in community settings [5]. Among them, nocturnal sleep is a typical daily lifestyle behavior, and sleep duration plays a significant role in hormone secretion and protein synthesis [6, 7], leading to alterations of muscle strength and physical performance. Thus, there is reason to believe that nocturnal sleep duration may be associated with sarcopenia. Numerous investigations have revealed a strong correlation between sarcopenia and the duration of nocturnal sleep [8–11]. However, there is some debate over whether short or long sleep duration has an effect on sarcopenia. In addition, the majority of previous studies employed a cross-sectional design. Thus, a longitudinal research is required to explore the effect of the duration of sleep on sarcopenia.

Midday napping is another typical daily lifestyle behavior. In China, over half of middle-aged and older people are reported to have regular naps [12]. A growing body of evidence indicated that midday napping is associated with physical, mental, and cognitive health in older adults [13–15]. Additionally, a systematic review showed that midday napping can improve physical and cognitive performance such as jumping, endurance, attention, and memory [16]. To date, there have been very limited studies to determine the association of midday napping with the presence of sarcopenia.

Therefore, the purpose of this study was to determine individual and combined associations of nocturnal sleep duration and midday napping with the incidence of sarcopenia in middle-aged and older people, utilizing data from a nationally representative population-based sample in China.

## 2. Methods

### 2.1. Setting and participants

This study used data from the 2011 and 2015 waves of the China Health and Retirement Longitudinal Study (CHARLS). The CHARLS survey aims to gather high-quality data on the demographic characteristics of middle-aged and older non-institutionalized Chinese individuals as well as information on their health state and functioning, families, employment and retirement, lifestyle behaviors, and health care and insurance. Comprehensive details about the CHARLS and a description of its methodology can be found on the official website (http://charls.pku.edu.cn/) and in earlier publications [17, 18].

**Fig. 1:**
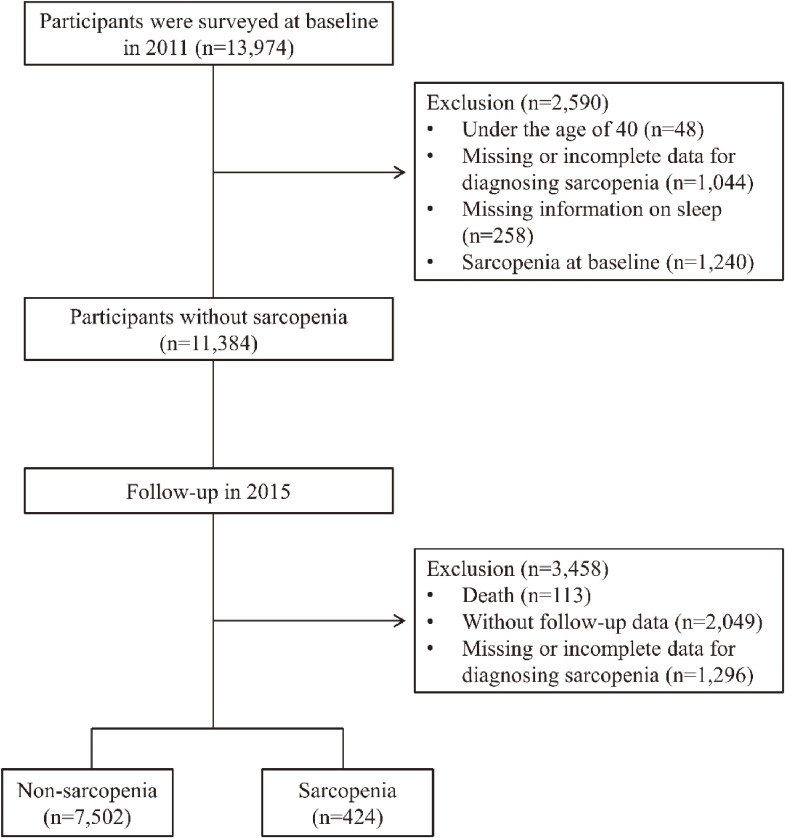
Flowchart of participants through this study

In the first wave of the survey, a total of 13,974 community-dwelling adults were enrolled. We excluded 2,590 individuals for the following reasons: (1) under the age of 40 (n = 48); (2) missing or incomplete data for diagnosing sarcopenia (n = 1,044); (3) missing information on sleep (n = 258); and (4) sarcopenia at baseline (n = 1,240). In total 11,384 participants without sarcopenia were included in the final study population. Nearly 4 years after the first wave, the third wave (2015) was conducted. The following were the exclusion criteria: (1) death (n = 113); (2) without follow-up data (n = 2,049); and (3) missing or incomplete data for diagnosing sarcopenia (n = 1,296). Finally, in total 7,926 middle-aged and older adults were employed in the study (Fig. 1). To determine the potential selection bias caused by missing data, variables such as gender, age, marital status, education level, and self-reported health were employed to compare the difference between the missing and observed data (Table S1).

### 2.2. Anthropometric measurements

Anthropometric measurements in the present study were comprised of body height and weight. Trained interviewers measured the participant’s height and weight directly. Subsequently, each participant’s body mass index (BMI) was determined by dividing their body weight by their squared height (kg/m^2^). According to the previous report, BMI was divided into <18.5 kg/m^2^ (underweight), <24.0 kg/m^2^ (normal weight), <28.0 kg/m^2^ (overweight), and ≥28.0 kg/m^2^ (obesity) [19].

### 2.3. Assessment of sleep behavior

At baseline, well-trained interviewers conducted a face-to-face questionnaire survey to obtain information on nocturnal sleep duration and midday napping. Respondents were asked to report how many hours they typically slept each night, and how long they had a nap during the past month. According to previous reports [19–21], nocturnal sleep duration was categorized into three groups: short sleepers (<6 h), normal sleepers (6–8 h), and long sleepers (>8 h); midday napping was coded as a dichotomous outcome (yes/no).

### 2.4. Assessment of sarcopenia

Based on the Asian Working Group for Sarcopenia 2019 (AWGS-2019) criteria [22], sarcopenia was diagnosed by a combination of the loss of muscle mass and the decrease in physical performance or muscle strength. Sarcopenia in our study was assessed using the following components: appendicular skeletal muscle mass (ASM), physical function, and muscle strength. We used a previously verified anthropometric equation to estimate the ASM for the Chinese population. Strong agreement was found between the anthropometric equation and the dual X-ray absorptiometry (the correlation coefficients ranging from 0.941 to 0.951) [23]. According to the anthropometric equation, ASM = 0.193 × body weight (kg) + 0.107 × body height (cm) − 4.157 × gender (male = 1, female = 2) − 0.037 × age (years) − 2.631. Similar to prior studies [24, 25], we adjusted the ASM by dividing it by the square of body height (ASM/Height^2^), and used the sex-specific lowest 20% of the ASM/Height^2^ as the cut-off to define the low muscle mass, which was 7.01 kg/m^2^ for males and 5.29 kg/m^2^ for females. 5-time chair stand test and handgrip strength were employed as measures of physical performance and muscle strength, respectively. According to the AWGS-2019 [22], we defined low physical performance as a finishing time of ≥12 seconds for the 5-time chair stand test, and defined low muscle mass as handgrip strength of <28 kg for men and <18 kg for women.

### 2.5. Covariates

Covariates in our study comprised lifestyle-related variables, health-related variables, and sociodemographic characteristics that were chosen based on prior research [25–27]. The sociodemographic characteristics covered age (40–49, 50–59, 60–69, and ≥70 years), gender (men and women), household registration type (agricultural, non-agricultural, and unified residence type), marital status (married and living with spouse, widowed, and others), and education levels (illiteracy, ≤primary school, middle school, and ≥high school). Lifestyle-related variables consisted of alcohol drinking frequency (>1/month, ≤1/month, and never drank) and smoking status (current smokers, former smokers, and never smoked). Health-related variables encompassed obesity levels (underweight, normal weight, overweight, and obesity), self-reported health (very good/good, fair, and poor/very poor), and chronic illness such as hypertension, diabetes, and dyslipidemia (yes and no).

### 2.6. Statistical analysis

Numbers with percentages were used to represent the study participants’ characteristics. We used Chi-square tests to determine the differences in these characteristics between sarcopenia and non-sarcopenia groups. Logistic regression analysis was performed to explore the association between nocturnal sleep duration, midday napping and the presence of sarcopenia at the follow-up years, with and without adjusting for gender, age, household registration type, marital status, education levels, smoking status, alcohol drinking frequency, obesity levels, self-reported health, hypertension, dyslipidemia, and diabetes. In the logistic regression analysis, the “6–8 h (normal sleepers)” for nocturnal sleep duration and the “no” for midday napping were set as the reference group, then the Odds ratios (OR) were calculated, together with their 95% confidence intervals (CI) based on two logistic regression models.

Logistic regression analysis was used to calculate the OR with 95% CIs for sarcopenia by the combined effect of nocturnal sleep duration and midday napping. In the combined analysis, normal sleepers without midday napping were set as the reference. Finally, to confirm the robustness of our findings, we conducted sensitivity analyses for participants without self-reported hypertension, dyslipidemia, or diabetes. SPSS version 26.0 (IBM Corp., Chicago, IL, USA) was employed to execute all the statistical analyses.

## 3. Results

There were 7,926 participants in the final analysis, and 424 of them had developed sarcopenia, representing an incidence of sarcopenia of 5.3% at 4-year follow-up. The differences in characteristics between sarcopenia and non-sarcopenia groups at the first wave were shown in Table 1. Compared to those without sarcopenia at baseline, those with new-onset sarcopenia were more likely to be underweight (21.5% vs. 2.7%, p < 0.001), older (35.6% vs. 8.2%, p < 0.001), and widowed (15.1% vs. 7.9%, p < 0.001), and had less education (39.6% vs. 24.0%, p < 0.001), bad or very bad self-reported health (61.8% vs. 49.9%, p < 0.001), and agricultural type (89.2% vs. 83.1%, p = 0.003).

**Table 1:** Baseline characteristics of participants based on the presence of sarcopenia

**Variables**	**Non-sarcopenia (n = 7,502)**	**Sarcopenia (n = 424)**	**p value**
Gender (n = 7,926)			0.615
Male	3,533 (47.1)	205 (48.3)	
Female	3,969 (52.9)	219 (51.7)	
Age (n = 7,926)			<0.001
40–49 years	1,768 (23.6)	18 (4.2)	
50–59 years	2,954 (39.4)	74 (17.5)	
60–69 years	2,162 (28.8)	181 (42.7)	
≥70 years	618 (8.2)	151 (35.6)	
Household registration type (n = 7,924)			0.003
Agricultural type	6,231 (83.1)	378 (89.2)	
Non-agricultural type	1,223 (16.3)	46 (10.8)	
Unified residence type	46 (0.6)	0 (0.0)	
Marital status (n = 7,926)			<0.001
Married and living with spouse	6,471 (86.3)	340 (80.2)	
Widowed	591 (7.9)	64 (15.1)	
Others	440 (5.9)	20 (4.7)	
Education levels (n = 7,926)			<0.001
Illiterate	1,798 (24.0)	168 (39.6)	
≤primary school	1,419 (18.9)	107 (25.2)	
Elementary school	1,773 (23.6)	94 (22.2)	
Middle school	1,712 (22.8)	40 (9.4)	
≥high school	800 (10.7)	15 (3.5)	
Smoking status (n = 7,925)			0.029
Current smokers	2,304 (30.7)	155 (36.6)	
Former smokers	622 (8.3)	37 (8.7)	
Never smoked	4,575 (61.0)	232 (54.7)	
Alcohol drinking frequency (n = 7,926)			0.150
>1/month	1,946 (25.9)	97 (22.9)	
≤1/month	606 (8.1)	28 (6.6)	
Never drank	4,950 (66.0)	299 (70.5)	
Obesity levels (n = 7,926)			<0.001
Underweight	200 (2.7)	91 (21.5)	
Normal weight	3,835 (51.1)	302 (71.2)	
Overweight	2,488 (33.2)	26 (6.1)	
Obesity	979 (13.0)	5 (1.2)	
Self-reported health (n = 7,923)			<0.001
Very good/good	1,231 (16.4)	50 (11.8)	
Fair	2,529 (33.7)	112 (26.4)	
Bad/very bad	3,739 (49.9)	262 (61.8)	
Hypertension (n = 7,881)			0.203
Yes	1,734 (23.3)	87 (20.6)	
No	5,724 (76.7)	336 (79.4)	
Dyslipidemia (n = 7,764)			0.210
Yes	694 (9.4)	32 (7.6)	
No	6,650 (90.6)	388 (92.4)	
Diabetes (n = 7,849)			0.196
Yes	407 (5.5)	17 (4.0)	
No	7,019 (94.5)	406 (96.0)	
Nocturnal sleep duration (n = 7,926)			<0.001
Short sleep duration	2,056 (27.4)	169 (39.9)	
Normal sleep duration	4,862 (64.8)	218 (51.4)	
Long sleep duration	584 (7.8)	37 (8.7)	
Midday napping (n = 7,926)			0.031
Yes	4,029 (53.7)	205 (48.3)	
No	3,473 (46.3)	219 (51.7)	

Based on two logistic regression models, the individual association between nocturnal sleep duration and midday napping and the incidence of sarcopenia was presented in Table 2. Short sleep duration (<6 h) was significantly linked with a higher risk of presence of sarcopenia compared to normal sleep duration (6–8 h), even after adjustment with confounders (OR: 1.50, 95% CI: 1.21–1.87). However, we have not found a significant relationship between the incidence of sarcopenia and long sleep duration (>8 h) from baseline to 4-year follow-up. Compared to participants without midday napping, those with midday napping had a lower risk of developing sarcopenia even after adjustment with confounders (OR: 0.78, 95% CI: 0.63–0.95). The results of calculation of effect modification in a stratified analysis by midday napping was described in Table S2. It was found that the main effect was inconsistent in the subgroup of adults with midday napping.

**Table 2:** Separate associations of nocturnal sleep duration and midday napping with sarcopenia

**Variables**	**Sarcopenia**	

	**OR (95% CI)**	**Adjusted OR^†^ (95% CI)**
Nocturnal sleep duration		
Normal sleep duration	1.00 (ref.)	1.00 (ref.)
Short sleep duration	1.83 (1.49–2.27)**	1.50 (1.21–1.87)**
Long sleep duration	1.41 (0.99–2.02)	1.15 (0.79–1.67)
Midday napping		
No	1.00 (ref.)	1.00 (ref.)
Yes	0.81 (0.66–0.97)*	0.78 (0.63–0.95)*

Abbreviations: OR, odds ratio; CI, confidential intervals

*p < 0.05, **p < 0.001

^†^Adjusted for gender, age, household registration type, marital status, education levels, smoking status, alcohol drinking frequency, obesity levels, self-reported health, hypertension, dyslipidemia, and diabetes

Table 3 illustrated the combined effects of nocturnal sleep duration and midday napping on the presence of sarcopenia. Compared to normal sleepers without midday napping, normal sleepers with midday napping had a lower risk of sarcopenia incidence (OR: 0.74, 95% CI: 0.56–0.99), while short sleepers without midday napping had a higher risk of sarcopenia incidence (OR: 1.37, 95% CI: 1.02–1.85). However, we discovered that short sleepers with midday napping did not have a remarkably increased risk of subsequent diagnosis of sarcopenia compared to normal sleepers without midday napping.

**Table 3:** Interaction between the duration of nocturnal sleep and midday naps on sarcopenia

**Nocturnal sleep duration**	**Midday napping**	

	**Non-nappers**	**Nappers**
Normal sleepers	1.00 (ref.)	0.74 (0.56–0.99)*
Short sleepers	1.37 (1.02–1.85)*	1.16 (0.84–1.62)
Long sleepers	0.94 (0.53–1.68)	0.99 (0.60–1.65)

Using logistic regression analysis to obtain odds ratio with 95% confidence interval after adjusting for gender, age, household registration type, marital status, education levels, smoking status, alcohol drinking frequency, obesity levels, self-reported health, hypertension, dyslipidemia, and diabetes. The reference is normal sleepers without midday napping.

*p < 0.05

We performed sensitivity analyses for participants without self-reported hypertension, dyslipidemia, or diabetes (Table S3). We found that the findings of the sensitivity analyses, which show associations of nocturnal sleep duration and midday napping with the incidence of sarcopenia, were largely consistent with the results in Table 2.

## 4. Discussion

The current study is, to the best of our knowledge, the first longitudinal study to determine the individual and combined associations of nocturnal sleep duration and midday napping with the presence of sarcopenia in middle-aged and older individuals, utilizing data from a national representative population-based sample in China. Our results indicated that short sleep duration is linked with an increased incidence of sarcopenia while midday napping is associated with a lower presence of sarcopenia. However, the adverse effect of short sleep duration on sarcopenia can be offset by midday napping. A sensitivity analysis showed that the association between nocturnal sleep duration and midday napping and the incidence of sarcopenia remained unchanged, after excluding individuals with self-reported hypertension, dyslipidemia, or diabetes.

The present study found an incidence of sarcopenia of 5.3% during the period between baseline and 4-year follow-up. The result is consistent with prior prospective studies, which reported that the incidence of sarcopenia was 1.06% per year among community-dwelling older adults in Japan [28], and 1.14% per year among the older population in English [29]. In our study, we have not observed significant differences in the presence of sarcopenia between males and females, which is in line with a previous study that demonstrated no remarkable differences in the prevalence of sarcopenia between genders [30]. Additionally, we have to admit that around 30 percent of the participants (3,458 of 11,384) were excluded from our study because of incomplete or missing data at the follow-up, which might result in sample selection bias. It was found that there were significant differences in age, household registration type, marital status, education levels, self-reported health, and obesity levels between the missing and observed groups. Nevertheless, the results of sensitivity analyses showed that our findings were robust after removing participants with self-reported hypertension, dyslipidemia, or diabetes.

The current study indicated that the incidence of sarcopenia was strongly associated with shorter sleep duration than longer sleep duration from baseline to 4-year follow-up. The current findings contradict previous studies that showed a remarkable relationship between long sleep duration and sarcopenia among older people [8, 31, 32]. In addition, other studies found a U-shape association, meaning that a greater probability of sarcopenia is associated with both short and long sleep hours [9, 33]. However, a majority of the previous studies were found to be based on a cross-sectional design. We offer potential explanations for why getting less sleep is linked to a higher risk of developing sarcopenia. First, it is known that certain growth-promoting hormones are secreted during nocturnal sleep, including those for protein synthesis, muscle repair, and tissue growth, and decreased sleep duration may inhibit the secretion of the growth-promoting hormones [6, 7]. Moreover, insulin resistance caused by sleep disturbances can lead to a decline in physical performance and muscle strength [34, 35].

Midday napping plays an important role in health promotion for human beings. Previous studies have demonstrated that midday napping is linked with a low likelihood of metabolism-related diseases and a high level of cognitive function [36, 37]. In the current study, we discovered that midday nappers had a reduced risk of developing sarcopenia when compared to non-midday nappers. The result is in line with a recently published study that suggested that daytime napping is one of the important factors affecting the risk of sarcopenia in older people [38]. The possible mechanism of midday napping on sarcopenia can be addressed from the viewpoint of physical performance. It is known that physical performance is a vital measure of the assessment of sarcopenia. Midday napping can induce a restorative process in the physiological system and lessen the oxidative damage in skeletal muscle triggered by short sleep or sleep deprivation [39], thereby improving physical performance [40].

Our findings suggested that short sleep duration may be a risk factor while midday napping is a protective factor for the incidence of sarcopenia. As a result, the negative impact of short sleep duration on sarcopenia can be offset by midday napping. The result is similar to a prior study that analyzed the associations of midday napping and nocturnal sleep duration with the risk of multimorbidity, which indicated that midday napping can compensate for the adverse impact of short nighttime sleep on multimorbidity [21]. In light of these, practitioners should consider changing lifestyle behaviors for those who have short sleep duration and have not regular naps to aid in sarcopenia prevention. Future research should investigate the sleep-sarcopenia mechanism, as well as whether modifying sleep behavior for short sleepers or non-midday nappers can result in a decreased risk of sarcopenia onset.

This study has multiple strengths. Compared to the closely related previous publications about nocturnal sleep duration and midday napping [8, 9, 21, 38], the use of a prospective cohort design, which can suggest a causal relationship between sleep behavior and the incidence of sarcopenia, is a major strength of this study. Another strength is that this study used a large-scale representative population-based sample in China.

There are several drawbacks to the current study. First, instead of using objective instruments, self-reported questionnaires were used to collect nocturnal sleep duration and midday napping. Second, despite adjusting for a range of relevant covariates, our study did not control other potential factors such as physical activity, dietary status, and caffeine intake that might influence the relationship between sleep behavior and sarcopenia. Third, instead of using dual X-ray absorptiometry or bioelectrical impedance analysis to assess muscle mass, we employed a previously established anthropometric formula that has been validated in the Chinese population [23]. Finally, our study’s 4-year follow-up was a relatively short period, which might result in a small change of health condition over the short observation time.

In conclusion, the present study showed the incidence of sarcopenia was 5.3% during the 4-year follow-up. Our findings imply that in middle-aged and older persons, short sleep duration is related to an increased incidence of sarcopenia while midday napping is linked with a decreased risk of sarcopenia. In addition, the adverse impact of short sleep duration on sarcopenia can be offset by midday napping. These findings are useful for those who have short sleep duration and have not regular naps to prevent or delay the onset of sarcopenia.

## Supplementary information

**Additional file 1:** **Table S1.** The basic characteristics of study participants at the follow-up according to the missing data. **Table S2.** Effect modification in a stratified analysis by midday napping. **Table S3.** Sensitivity analyses for participants without self-reported hypertension, dyslipidemia, or diabetes.

## Declarations

### Availability of data and materials

The study’s supporting data are accessible at http://charls.pku.edu.cn/en/index.htm, through Peking University Open Research Data.
