# Supplementary material for: Association between nocturnal sleep duration and midday napping and the incidence of sarcopenia in middle-aged and older adults: a 4-year longitudinal study
Source: Environ Health Prev Med. 2024 May 17;29:29. doi: 10.1265/ehpm.24-00046 (PMC11157246; doi:10.1265/ehpm.24-00046)
Supplement: Supplementary file 1 — Additional file 1: Table S1. The basic characteristics of study participants at the follow-up according to the missing data. Table S2. Effect modification in a stratified analysis by midday napping. Table S3. Sensitivity analyses for participants without self-reported hypertension, dyslipidemia, or diabetes. [file ehpm-29-029-s001.docx]

**Supplementary material**

**Table Legend**

**Table S1.** The basic characteristics of study participants at the follow-up according to the missing data

**Table S2.** Effect modification in a stratified analysis by midday napping

**Table S3.** Sensitivity analyses for participants without self-reported hypertension, dyslipidemia, or diabetes

| **Table S1.** The basic characteristics of study participants at the follow-up according to the missing data | | | |
| --- | --- | --- | --- |
| Variables | Missing data (n=3,458) | Observed data (n=7,926) | p value |
| Gender (n=11,383) |  |  | 0.539 |
| Male | 1,652 (47.8) | 3,738 (47.2) |  |
| Female | 1,805 (52.2) | 4,188 (52.8) |  |
| Age (n=11,353) |  |  | < 0.001 |
| 40-49 years | 754 (22.0) | 1,786 (22.5) |  |
| 50-59 years | 1,234 (36.0) | 3,028 (38.2) |  |
| 60-69 years | 852 (24.9) | 2,343 (29.6) |  |
| ≥70 years | 587 (17.1) | 769 (9.7) |  |
| Household registration type (n=11,376) |  |  | < 0.001 |
| Agricultural type | 2,617 (75.8) | 6,609 (83.4) |  |
| Non-agricultural type | 820 (23.8) | 1,269 (16.0) |  |
| Unified residence type | 15 (0.4) | 46 (0.6) |  |
| Marital status (n=11,377) |  |  | < 0.001 |
| Married and living with spouse | 2,709 (78.5) | 6,811 (85.9) |  |
| Widowed | 391 (11.3) | 655 (8.3) |  |
| Others | 351 (10.2) | 460 (5.8) |  |
| Education levels (n=11,370) |  |  | < 0.001 |
| Illiterate | 847 (24.6) | 1,966 (24.8) |  |
| ≤primary school | 601 (17.5) | 1,526 (19.3) |  |
| Elementary school | 790 (22.9) | 1,867 (23.6) |  |
| Middle school | 757 (22.0) | 1,752 (22.1) |  |
| ≥high school | 449 (13.0) | 815 (10.3) |  |
| Smoking status (n=11,207) |  |  | 0.273 |
| Current smokers | 994 (30.3) | 2,459 (31.0) |  |
| Former smokers | 250 (7.6) | 659 (8.3) |  |
| Never smoked | 2,038 (62.1) | 4,807 (60.7) |  |
| Alcohol drinking frequency (n=11,347) |  |  | < 0.001 |
| >1/month | 758 (22.2) | 2,043 (25.8) |  |
| ≤1/month | 295 (8.6) | 634 (8.0) |  |
| Never drank | 2,368 (69.2) | 5,249 (66.2) |  |
| Obesity levels (n=10,518) |  |  | < 0.001 |
| Underweight | 181 (7.0) | 291 (3.7) |  |
| Normal weight | 1,393 (53.7) | 4,137 (52.2) |  |
| Overweight | 756 (29.2) | 2,514 (31.7) |  |
| Obesity | 262 (10.1) | 984 (12.4) |  |
| Self-reported health (n=11,349) |  |  | 0.031 |
| Very good/good | 616 (18.0) | 1,281 (16.2) |  |
| Fair | 1,082 (31.6) | 2,641 (33.3) |  |
| Bad/very bad | 1,728 (50.4) | 4,001 (50.5) |  |
| Hypertension (n=11,272) |  |  | 0.351 |
| Yes | 811 (23.9) | 1,821 (23.1) |  |
| No | 2,580 (76.1) | 6,060 (76.9) |  |
| Dyslipidemia (n=11,088) |  |  | < 0.001 |
| Yes | 237 (9.4) | 726 (9.4) |  |
| No | 3,087 (90.6) | 7,038 (90.6) |  |
| Diabetes (n=11,218) |  |  | 0.899 |
| Yes | 180 (5.3) | 424 (5.4) |  |
| No | 3,189 (94.7) | 7425 (94.6) |  |

| **Table S2.** Effect modification in a stratified analysis by midday napping | | |
| --- | --- | --- |
| Nocturnal sleep duration | Midday napping | |
|  | No | Yes |
| Normal sleepers | 1.00 (ref.) | 1.00 (ref.) |
| Short sleepers | 1.38 (1.03 - 1.85)^*^ | 1.28 (0.91 - 1.80) |
| Long sleepers | 0.96 (0.54 - 1.70) | 1.08 (0.63 - 1.84) |
| Using logistic regression analysis to obtain odds ratio with 95% confidence interval after adjusting for gender, age, household registration type, marital status, education levels, smoking status, alcohol drinking frequency, obesity levels, self-reported health, hypertension, dyslipidemia, and diabetes  ^*^ p < 0.05 | | |

| **Table S3.** Sensitivity analyses for participants without self-reported hypertension, dyslipidemia, or diabetes | | |
| --- | --- | --- |
| Variables | Sarcopenia | |
|  | OR (95% CI) | Adjusted OR^†^ (95% CI) |
| Nocturnal sleep duration |  |  |
| Normal sleep duration | 1.00 (ref.) | 1.00 (ref.) |
| Short sleep duration | 1.82 (1.43 - 2.32)^***^ | 1.43 (1.11 - 1.84)^**^ |
| Long sleep duration | 1.25 (0.81 - 1.93) | 1.04 (0.66 - 1.63) |
| Midday napping |  |  |
| No | 1.00 (ref.) | 1.00 (ref.) |
| Yes | 0.79 (0.63 - 0.98)^*^ | 0.78 (0.61 - 0.98)^*^ |
| Abbreviations: OR, odds ratio; CI, confidential intervals | | |
| ^*^ p < 0.05, ^**^ p < 0.01, ^***^ p < 0.001 | | |
| ^†^ Adjusted for gender, age, household registration type, marital status, education levels, smoking status, alcohol drinking frequency, obesity levels, and self-reported health | | |
